# Development and validation of the Pachinko/Pachi-Slot Playing Ambivalence Scale

**DOI:** 10.1186/s40405-017-0023-6

**Published:** 2017-05-12

**Authors:** Yasunobu Komoto, Akiyo Shoun, Kumiko Akiyama, Akira Sakamoto, Taku Sato, Naoyuki Nishimura, Kikunori Shinohara, Hitoshi Ishida, Nobuo Makino

**Affiliations:** 1 Yoshino Hospital, 2252, Zushi-tyo, Machida City, Tokyo 1940203 Japan; 2 Graduate School of Humanities and Sciences, Ochanomizu University, Bunkyō, Japan; 3 The Nikkoso Research Foundation for Safe Society, Tokyo, Japan; 4 Department of Psychology, Ochanomizu University, Bunkyō, Japan; 5 Naruse Mental Clinic, Machida City, Japan; 6 NPO Recovery Support Network, Tokyo, Japan; 7 Tokyo University of Science Suwa, Chino, Japan; 8 Japan Women’s University, Kawasaki, Japan

**Keywords:** Gambling disorder, Pachinko/pachi-slot playing disorder, Ambivalence scale, DSM5, Severity

## Abstract

**Background:**

A scale aimed at measuring ambivalence among people with pachinko/pachi-slot playing disorder, the Pachinko/Pachi-Slot Playing Ambivalence Scale (PPAS), was developed and its reliability and validity ascertained.

**Methods:**

A total of 522 participants (average year: 48.0) who were residing in Tokyo Metropolitan Area, and had played pachinko within the previous year completed questions relating to demographics, four gambling-related scales (including South Oaks Gambling Screen) and two general ambivalence scales (including Ambivalence over Emotional Expressiveness Questionnaire).

**Results:**

Internal consistency (α = 0.87) and test–retest reliability (r = 0.66) were confirmed. The PPAS’s score was associated with each related scale’s score (r = 0.37–0.62).

**Conclusions:**

The PPAS was shown to be consistent with previous scales and useful in clinical settings.

## Background

The lifetime prevalence of gambling disorders around the world has been reported to be about 1.5% (Gowing et al. 2015), similar to that of schizophrenia and bipolar disorder. Not only gambling disorder promotes depression and suicide (Petry and Kiluk 2002), but it has been linked to social problems such as child abuse and severe indebtedness (Grant et al. 2010). Therefore, the development of intervention guidelines based on appropriate diagnostic and assessment measures has become a pressing issue.

Existing gambling disorder assessment scales can broadly be divided into: (a) scales for evaluating treatment effectiveness by measuring principal symptoms such as a craving and (b) diagnostic scales providing a comprehensive assessment of problems; for example, in cognition, behavior, and interpersonal relationships. The former type includes the Gambling Symptom Assessment Scale (G-SAS) (Kim et al. 2009) and the Yale-Brown Obsessive Compulsive Scale-modified for Pathological Gambling (PG-YBOCS) (Pallanti et al. 2005). The latter type includes assessment instruments such as the Diagnostic and Statistical Manual of Mental Disorders-5 (DSM-5) (American Psychiatric Association 2013), the South Oaks Gambling Screen (SOGS) (Lesieur and Blume 1987), the Alberta Gaming Research Institute (AGRI) Short Version (Volberg and Williams 2011), the Problem Gambling Severity Index (PGSI) (Ferris and Wynne 2001), and the Lie–Bet Screen (Johnson et al. 1997). These diagnostic scales (b) enable assessment of problem severity, based on several different pathological concepts in a given case. In other words, these scales focus not on a single pathological concept but on multiple pathological concepts such as psychopharmacology of substance use disorder, psychodynamics, and interpersonal model (Stinchfield 2013). For example, the nine items of DSM5 consist of four different pathological concepts, namely psychopharmacology, psychodynamics, interpersonal and socio-economics model. Similarly, Lie–Bet Screen consists of two concepts, interpersonal model and psychopharmacology. On the other hands, scales, which focus on a single pathological concept, have been developed. For example, the Gambling Functional Assessment-Revised measures psychopharmacological dependency, namely positive and negative reinforcement such as resistance and withdrawal (Weatherly et al. 2011); whereas Gamblers’ Beliefs Questionnaire measures cognitive distortions such as neglecting of randomness (Steenbergh et al. 2002).

Although various useful scales for gambling disorders have been developed, it is not clear if these scale measure core symptoms that explain the basic mechanism of gambling disorder.

The importance of the concept of ambivalence, being that “alcoholics simultaneously want to quit and do not want to quit,” has been raised in substance addiction research (Walker et al. 2011), because it has been found to be a predictor of relapse in drinking behavior (including heavy drinking) and drug abuse (Lipkus et al. 2001; Oser et al. 2010), and of relapse in ex-smokers (Menninga et al. 2011). Additionally, ambivalence regarding alcoholism is an important determinant of drinking behavior in the same way that craving for alcohol is (Dawn et al. 2014). In many instances, ambivalence acts as an inhibitory factor in recovery (Armitage 2003).

Bleuler, who first coined the concept of ambivalence, encompassed two different ideas. He pointed out that ambivalence can be a symptom of pathology because two opposite psychological phenomena continue to exist in parallel, or it can have the common meaning of tying different psychological phenomena together via consistent values (Bleuler 1914/1997; Hitomi 2011). Therefore, in the assessment of ambivalence, these two aspects must be covered. The former is a psychopathological finding, which reveals failure of solution to conflicts, such as “parallel existence of expectation, emotion, and reason” (Bleuler 1914/1997, p. 136). On the other hand, the latter reveals a self-oriented, rational response after conflictive behaviors, such as regret.

When assessing ambivalence, one either assesses structural ambivalence by differentiating and measuring two conflicting factors such as feelings, thoughts and behaviors, or subjective ambivalence by assessing the psychological state that arises when two conflicting factors coexist (Priester and Petty 1996). For example, Drinking Ambivalence Scale (DAS) (Dawn et al. 2014) measures structural ambivalence; whereas, General Ambivalence Scale (Thompson et al. 1995) and a six-item ambivalence scale for smoking (Lipkus et al. 2001) focus on subjective ambivalence. However, Conner and Sparks (2002) report there is a significant correlation between two assessment methods.

Currently, no scale has been developed for this concept in gambling. Thus, in this study, we developed a scale to measure ambivalence towards gambling behavior. In Japan, pachinko/pachi-slot playing disorder accounts for nearly 90% of all gambling disorders (Toyama et al. 2014; Komoto 2014). Pachinko and pachi-slot constitute private gambling involving use of a device similar to a recreational arcade game. There are many pachinko/pachi-slot parlors in every downtown area in Japan. Therefore, we first developed the Pachinko/Pachi-Slot Playing Ambivalence Scale (PPAS), and tested its reliability and validity. Improving classification (severity/subtype) and prediction of prognosis are not the only reasons for the incorporation of ambivalence into the diagnosis and treatment of gambling disorder. A better understanding of ambivalence by those providing support to people with gambling disorders may enhance their understanding of the recovery process that may face frequent relapses. In addition, for the gambler, better understanding could provide an opportunity to think about the cravings that drive his/her urge to gamble (Komoto and Sato 2014).

## Methods

### Participants

#### Initial survey

Using an online survey company, we recruited members registered as internet-shopping customers residing in Tokyo, in Saitama, Chiba, or in Kanagawa Prefectures, who had played pachinko or pachi-slot within the previous year. A total of 522 people agreed to participate in the survey, comprising the ambivalence scale and an impression management subscale (Paulhus 1991).

Of the 522 participants, 446 (85.4%) were men and most were in their 40 s (35.8%) or 50 s (28.0%). The majority of the participants were individuals who had at least graduated from college (77.4%), lived with a family (not be single) (72.8%), and had an annual household income of ¥4–10 million (60.4%; the so-called “middle economical class” in Japan).

#### Retest survey

We used the same online survey company and asked the 522 from the initial survey to participate again in the survey. Sixty-six participants (12.6%) of the original sample (n = 522) agreed to answer the retest questionnaire.

### Measures

#### Playing frequency, duration, and expenditure

The frequency of playing pachinko/pachi-slot and expenditure (i.e., “money lost”) over the previous 12 months were measured. Responses regarding frequency were rated on a 9-point scale from 1 (less than once a year) to 9 (more than 4 times a week). Playing duration was measured through average playing duration per day, on an 8-point scale, from 1 (less than 1 h) to 8 (8 h or more). Expenditure on playing was measured through the average amount of money lost per month, on a 7-point scale from 1 (I do not lose) to 7 (more than ¥200,000). A response indicating the lowest expenditure in this regard was allocated 1 point. Responses to “I do not lose” were merged with those to “less than ¥10,000,” with either option assigned 1 point.

#### The Pachinko/Pachi-Slot Playing Ambivalence Scale (PPAS)

Several congresses were held by three psychiatrists and four researchers specializing in psychology, education, neuroscience, and sociology to develop items of the PPAS. All psychiatrists were specialists of addictive disorders. Three researchers were experienced researchers of universities, and one researcher of sociology was also a specialist of statistics. During this process, the six-item ambivalence scale for smoking (Lipkus et al. 2001) and other existing ambivalence-related scales were used for reference (Dawn et al. 2014; King and Emmons 1990; Lipkus et al. 2005; Nagano et al. 2001). This six-item ambivalence scale for smoking consisted of the following six self-descriptive assessments: (1) “I have strong feelings both for and against smoking”; (2) “I have conflicting thoughts and feelings about smoking; sometimes I think that smoking is good, while at other times I think that it is bad”; (3) “My gut feeling and my thoughts do not seem to agree on whether I should smoke”; (4) “I find myself feeling torn between wanting and not wanting to smoke”; (5) “My gut feeling about whether to smoke agrees perfectly with what my mind tells me” (a reversed question); and (6) “I have equally strong reasons for wanting and not wanting to smoke.” Although this scale has good internal consistency and prognosis-predictive ability, some items are abstract, with terms such as “good,” “bad,” and “gut.” Therefore, we created the PPAS, with more concrete and clear expressions and consisting of two factors and nine items, as follows: three items concerning “regret” (e.g. “After losing money playing pachinko/pachi-slot, I wished that I had spent it on something delicious to eat.”) and six items concerning “parallel expectations, emotions, and reasons” (e.g. “When I was playing pachinko or pachi-slot, I felt both happy and distressed or “In my mind, I want to quit playing pachinko/pachi-slot and at the same time, I want to play.”).

The rating was on a 4-point scale, as follows: (1) “Not true,” (2) “Maybe not true,” (3) “Maybe true,” and (4) “True.” The total score range was 9–36. Participants were asked to consider the questions regarding their gambling behavior only in the previous 12 months.

#### Factor analysis of the PPAS

We conducted an exploratory factor analysis (EFA) of nine items of the PPAS. Because all factors were considered dependent upon each other, the factor solution was sought after Promax rotation, which is an oblique rotation. The number of factors was determined through the scree plot (Cattell 1966). To create subscales of the PPAS, we extracted items for each subscale if they yielded a loading of >0.3 on a particular factor, but of <0.3 on other factors.

Thereafter, using maximum likelihood estimation, some factor structures including one derived from the EFA were confirmed through confirmatory factor analysis (CFA) among the same group of 522 participants. The fit of each data model was examined through the goodness of fit index (GFI), adjusted goodness of fit index (AGFI), comparative fit index (CFI) and root mean square error of approximation (RMSEA). According to conventional criteria, GFI > 0.9, AGFI > 0.9, CFI > 0.95, and RMSEA < 0.08 indicate an acceptable fit (Schermelleh-Engel et al. 2003).

Additionally Cronbach’s alpha for the hypothesized subscales was calculated to examine the internal reliability of the PPAS. The acceptable standards for alpha values are ranging from 0.70 to 0.95. (Tavakol and Denneck 2011).

#### Scales used to test concurrent validity

To examine concurrent validity, we used both the general ambivalence and gambling scales.

##### General ambivalence scales

The Short Interpersonal Reactions Inventory (SIRI)—Japanese version (Grossarth-Maticek and Eysenck 1990; Nagano et al. 2001).

This scale is a self-administered scale, with its reliability for use in Japan having been confirmed. Participants were required to answer “Yes”/“No” items related to the “ambivalent object-dependent type” characterized by an ambivalent attitude. We selected only the most representative three items to shorten a questionnaire. The items were as follows: “I alternate to a great degree between positive and negative evaluation of people and situations”; “With people I love, I oscillate between them at a great distance to stifling dependence, and from stifling dependence to excessive distancing”; “As soon as someone becomes emotionally close to me, I tend to place contradictory demands on them, such as ‘Don’t ever leave me’ and ‘Get away from me.’” The score range for these items was from 3 to 6.

Ambivalence over Emotional Expressiveness Questionnaire (AEQ) (King and Emmons 1990).The AEQ is a self-administered scale consisting of 28 items, used to assess ambivalence in emotional expressiveness in interpersonal relations. Since there is no Japanese version, the scale’s reliability and validity have not been confirmed for use in Japan. To shorten a questionnaire, we selected the following four items, referring to Cronbach’s alpha, which were rated on a 5-point scale, e.g. 0 (strongly disagree) to 4 (strongly agree): “Often I find that I cannot tell others how much they really mean to me”; “I want to tell someone people when I love them, but it is difficult to find the right words”; “After expressing anger at someone, it bothers me for a long time”; and “I feel guilty after having expressed anger at someone.” The possible range of the scale scores varied from 0 to 16.

#### Measures of gambling disorder

In all of the gambling disorder’s items, the word “gambling” was replaced with the word “pachinko/pachi-slot playing.”

##### The Diagnostic and Statistical Manual of mental Disorders-5 (DSM-5)

Nine items were adapted from the Japanese version of the nine DSM-5 criteria for gambling disorder (American Psychiatric Association 2013). The original DSM-5 wording was changed to make the items relevant to the questionnaire context and easier for respondents to understand. For example, “In a 12-month period…” became “In the last 12 months….” Moreover, the criteria regarding experiences of emotions and problems were expressed in a “Yes”/”No” question format. Upon scoring, a “Yes” response was assigned one point, so that the total possible score range was 0–9. We used the DSM-5 severity levels and simply translated the number of criteria met into points; in other words, mild severity was 4–5 points, moderate was 6–7 points, and severe was 8–9 points.

##### The South Oaks Gambling Screen (SOGS)

We translated 19 of the SOGS’s 20 items (Lesieur and Blume 1987) into Japanese. We omitted translating the item concerning the writing of bad checks to cover gambling debt, as it is not relevant in the Japanese context. The answers were scored on the basis of Lesieur and Blume’s (1987) method. Scores were determined by adding up the number of questions which show an “at risk” response. Nineteen questions were scored 0 or 1. Therefore the score range was 0–19.

##### The Problem Gambling Severity Index (PGSI)

The PGSI is a 9-item scale requiring the respondent to think about the past 12 months (Ferris and Wynne 2001), with questions such as, “Have you bet more than you could really afford to lose?,” using a scale of 1 (“never”) to 4 (“almost always”). The score range was 9–36.

##### The Alberta Gaming Research Institute (AGRI) Short Screen

The AGRI Short Screen is a 5-item scale requiring the respondent to think about the past 12 months (Volberg and Williams 2011) and reply with “Yes” or “No” to questions such as, “Would you say you have been preoccupied with gambling?” (as adapted for this study). A score of 1 was assigned to each “Yes” answer. The score range was 0–5.

#### A gambling dependency diagnosis status

Participants were asked the question, “During the past year, have you ever been told by a medical or treatment support facility that you suffer from gambling dependence?,” to which they were to respond “Yes,” “No,” or “Don’t wish to answer.” A score of 1 was assigned for “Yes,” and 0 for “No”; “Don’t wish to answer” was treated as missing data. The score range was 0–1.

#### Social desirability

In order to check for the possibility of responses having been biased by the respondents’ desire for social approval, we included the 12-item Impression Management subscale from the Balanced Inventory of Desirable Responding. Respondents were asked to rate each item (e.g., “I sometimes tell lies if I have to.”) on a scale of 1–4, with 1 indicating “Not true” and 4 indicating “Very true” (Paulhus 1991; Tani 2008). The score range was 12–48.

### Procedure

Data for the initial and retest surveys were collected via self-administered online questionnaires at an interval of approximately two weeks in February 2015.

#### Statistical analysis

To determine validity, we observed the correlations between the PPAS and the scales presented in the preceding sub-sections, playing frequency, the gambling dependency diagnosis status, and social desirability by using Pearson’s correlation coefficients. To test for reliability, we observed the correlations between the initial data and the retest data. Significance was set at *p* < 0.05.

#### Ethics

The study procedures were carried out in accordance with the Declaration of Helsinki. The Institutional Review Board of Ochanomizu University approved the study. All subjects were informed about the study and all provided informed consent. Participant data were treated as strictly confidential and anonymous.

## Results

### Playing frequency and expenditure

With regard to playing frequency, 23.2% of the participants played 2–3 times per week, followed by 21.5%, who played 2–3 times per month. Moreover, 21.0% had played ≤4 times within the past year and 5.4% had played ≥4 times per week. Most of the participants (n = 150, 28.7%) played for 2–3 h, followed by 22.6%, who played for 3–4 h. In total, 8.0% played for ≥6 h. With regard to monthly expenditure, 28.0 and 26.2% of the participants reported losing less than ¥10,000 and ¥20,000–¥50,000, respectively. However, 6.3% reported never losing and 5.3% reported a loss of ¥100,000 or more.

### PPAS

The mean total score (SD) of PPAS was 21.3 (6.25). More “Very true” or “True” responses were given for “regret” (26.4–33.1%), as compared to “paralleling” (5.7–18.2%).

#### EFA

The entire log-transformed items of the PPAS were entered into an EFA. This suggested a two-factor structure. Factor 1 was loaded by three items (1–3), which expressed “regret” for gambling. Regret is a conflictive reaction after an inconsistent behavior, because feeling regret meant that gamblers recognized that the food, goods, and friendship were more important to them than gambling was. Factor 2 was loaded by six items that expressed coexistence of opposite thoughts, feelings and motivations, e.g. “a desire to gamble and a desire to quit gambling”.

A two-factor structure was suggested in PPAS (Table 1).

**Table 1:** Exploratory factor analysis (EFA) for the PPAS

	Please rate these statements thinking about the past 12 months	Factor	
		1	2
	1. After losing money playing pachinko or pachi-slot, I wished that I had spent it on something delicious to eat	*0.947*	−0.082
Regret	2. After losing money playing pachinko or pachi-slot, I wished that I had used it to buy something I wanted	*0.888*	0.03
	3. After losing money playing pachinko or pachi-slot, I wished that I had used the money to go out with my sweetheart or a friend	*0.862*	0.006
	4. When I am playing pachinko/pachi-slot, thoughts run through my mind that I could get rich, but also that I could go bankrupt	−0.026	*0.771*
	5. When I am playing pachinko/pachi-slot, thoughts run through my mind that the people I care about might praise me for playing, or that they might reproach me for playing	−0.059	*0.749*
Parallel	6. When I was playing pachinko or pachi-slot, I felt both happy and distressed	0.057	*0.749*
	7. In my mind, I want to quit playing pachinko/pachi-slot and at the same time, I want to play	0.205	*0.547*
	8. The reason I play pachinko/pachi-slot is to win and also to lose	−0.116	*0.523*
	9. The reason I play pachinko/pachi-slot changes with the moment	0.126	*0.491*

N = 522 (principal component analysis with promax rotation)

Italics mean each two factor group. Factor 1 consists of item 1–3, and factor 2 consists of item 4–9

#### CFA

Three models were tested. The first model to be examined was a one-factor model in which all nine items were predicted to load onto a single factor generally reflecting the ambivalence of disordered gamblers. The analysis showed that the single-factor solution was not a good fit for the data. All fitted indices were less than the acceptable value of 0.9.

The second model to be examined was the two-factor model, which was extracted in the EFA. Although this model was a better fit than the one-factor model, the fit was not adequate, as the AGFI value was less than 0.9. Moreover, the RMSEA value fell outside the accepted value, further suggesting that the two-factor model was not the best fit for the data.

The third model was the four-factor model, which was assumed logically. The parallel factor, which was one factor of the two-factor model, could be divided into three sub-factors, namely, parallel expectation (items 4–5; e.g. “when I am playing pachinko/pachi-slot, thoughts run through my mind that I could get rich, but also that I could go bankrupt.”), parallel emotion (items 6–7; e.g. “In my mind, I want to quit plying pachinko/pachi-slot and at the same time, I want to play.”), and parallel reasons (items 8–9; e.g. “The reason I play pachinko/pachi-slot is to win and also to lose.”). The analyses showed that this four-factor model was a good fit for the data, as all fit indices were greater than 0.9 (GFI = 0.967; AGFI = 0.929; CFI = 0.975). Furthermore, the RMSEA value was in the accepted range (0.074).

Three models were tested and four-factor model was a best fit for the data, as all fit indices were greater than 0.9 Table 2.

**Table 2:** CFA of the PPAS

	χ2	Degrees of freedom	GFI	AGFI	CFI	RMSEA
4-factor model	81.058	21	0.967	0.929	0.975	0.074
2-factor model	186.299	26	0.921	0.863	0.934	0.109
1-factor model	713.293	27	0.703	0.505	0.718	0.221

### Reliability

#### Internal consistency (Cronbach’s alpha)

Internal consistency coefficients (Cronbach’s alpha) for the overall scale and each factor were as follows: α = 0.87 for the total score, α = 0.92 for “regret,” α = 0.79 for “parallel expectations,” α = 0.80 for “parallel emotions,” and α = 0.48 for “parallel reasons.”

#### Test–retest reliability (n = 66)

Pearson’s correlation coefficients for the initial and retest scores were 0.66 for the total score, 0.62 for “regret,” 0.42 for “parallel expectations,” 0.56 for “parallel emotions,” and 0.50 for “parallel reasons.” All were significant at *p* < 0.01.

### Validity

#### Correlations with related scales

Scales related to the PPAS showed significant positive correlations with the PPAS and with each of its subscales.

Total score and sub score of PPAS correlated with other gambling- and ambivalent- related scales Table 3.

**Table 3:** The PPAS’s correlations with related scales

	SIRI	AEQ-G	SOGS	DSM-5	AGRI	PGSI
Mean total score	0.37	0.43	0.58	0.62	0.54	0.43
Regret	0.18	0.33	0.38	0.4	0.36	0.27
Parallel expectations	0.39	0.32	0.53	0.54	0.46	0.44
Parallel emotions	0.34	0.38	0.58	0.61	0.53	0.43
Parallel reasons	0.27	0.28	0.33	0.41	0.34	0.22

All correlation coefficients were significant at *p* < 0.01

Next, we divided the participants into four groups according to the DSM-5 severity score (none, mild, moderate, and severe) and compared the mean PPAS scores across severity groups. For the procedure, we performed a one-way analysis of variance on the means for the four groups and found a significant between-group effect [*F*(3, 518) = 78.58, *p* < 0.001]. Differences between the mean values were then assessed using the Bonferroni comparison procedure. The results showed that the scores increased with severity.

Mean total score of PPAS correlated with severity assessed by DSM5 Table 4.

**Table 4:** A comparison of PPAS total scores by DSM5-severity group

Severity classification	N	Mean	SD	SE	Mean at 95% CI		Mnimum value	Maximum value
					Lower limit	Upper limit		
None	349	18.9a	5.66	0.303	18.3	19.5	9	36
Mild	84	25.4b	3.98	0.435	24.5	26.2	12	34
Moderate	57	25.8bc	3.91	0.518	24.8	26.8	18	36
Severe	32	28.7c	4.62	0.816	27	30.4	18	36
Total	522	21.3	6.25	0.274	20.7	21.8	9	36

Means followed by the same letter do not differ significantly (*p* = 0.05)

#### Correlations with a gambling dependency diagnosis status

The correlations of a gambling dependency diagnosis status with the PPAS and its subscales were as follows: 0.21 for the total score, 0.09 for “regret,” 0.21 for “parallel expectations,” 0.16 for “parallel emotions,” and 0.24 for “parallel reasons.” The correlation for “regret” was significant at *p* < 0.05, and the rest at *p* < 0.01.

#### Correlations with playing frequency and expenditure (convergent validity)

There were significant positive correlations (*p* < 0.01) between the total PPAS score and “frequency” (0.20), “playing duration” (0.17), and “money lost” (0.37).

#### Discriminant validity

##### Correlations with the social desirability scale

Significant negative correlations (*p* < 0.01) were found between social desirability and the total PPAS score (−0.30), “regret” (−0.20), “parallel expectations (−0.33), “parallel emotions” (−0.22), and “parallel reasons” (−0.18).

##### Correlations with demographic factors

No significant differences were found in the total PPAS score according gender, education level (higher or lower than college-graduate level) and family structure (single or not single). Similarly, no significant results were found for the correlation between household income and the total PPAS and subscale scores. Significant negative correlations were found between age group and the total PPAS score and each sub score (*p* < 0.05).

No significant differences were found in the total PPAS score according gender Table 5.

**Table 5:** The PPAS’s difference concerning demographic factors (gender)

t-test/mean score	Male (n = 446)	Female (n = 76)
Total	21.4	20.4
Regret	8.48	8.24
Parallel expectations	3.79	3.63
Parallel emotions	4.79	4.53
Parallel reasons	4.37	4.04

No significant differences were found in the total PPAS score according education level Table 6.

**Table 6:** The PPAS’s difference concerning demographic factors (education)

t-test/mean score	Over colleage (n = 404)	Under high school (n = 118)
Total	21.3	21.2
Regret	8.48	8.31
Parallel expectations	3.76	3.79
Parallel emotions	4.74	4.79
Parallel reasons	4.31	4.34

No significant differences were found in the total PPAS score according family structure Table 7.

**Table 7:** The PPAS’s difference concerning demographic factors (family structure)

t-test	With a family (n = 380)	Single (n = 142)
Total score	21.1	21.8
Regret	8.33	8.74
Parallel expectations	3.69	3.96
Parallel emotions	4.74	4.78
Parallel reasons	4.31	4.33

Significant negative correlations were found between age group and the PPAS score (*p* < 0.05). On the other hand, no significant results for the correlation were found between household income and the PPAS Table 8.

**Table 8:** The PPAS’s correlations with demographic factors (household income and age group)

Correlation test	Household income	Age group*
Total score	−0.05	−0.2
Regret	−0.04	−0.15
Parallel expectations	−0.08	−0.3
Parallel emotions	−0.05	−0.09
Parallel reasons	−0.01	−0.09

* Significant correlation: *p* < 0.05

## Discussion

### The PPAS’s reliability

The scale’s reliability was confirmed. Despite the low Cronbach’s alpha value for “parallel reasons,” at 0.48, those for the total scores and other three factors’ scores were 0.79–0.92, demonstrating the scale’s high internal consistency. Moreover, the test–retest correlation coefficients were 0.64 for the overall scale and between 0.42 and 0.62 for the subscales. Regarding parallel reasons, item 9 has a wider concept beyond ambivalence. Namely, changing the reason is not always associated with ambivalent attitude. Therefore the factor “parallel reasons” demonstrated the relative low internal consistency.

### The PPAS’s validity

#### Construct validity

Results revealed that the four factors model reflected the classical distinctions drawn by Bleuler in defining regret.

#### Concurrent validity

There were significant positive correlations (0.37–0.62) between the total PPAS score and those of the related general scales (SIRI, AEQ) and gambling scales (SOGS, DSM-5, AGRI Short Screen, PGSI). Moreover, the correlations for the parallel factors tended to be higher than those for the regret factor. Additionally, there were small but significant positive correlations between the gambling dependency diagnosis status and the PPAS’s total score and its paralleling-factor scores. Thus, the PPAS’s concurrent validity was confirmed.

#### Convergent validity

The PPAS scores showed small to medium positive correlations with playing frequency and expenditure. In particular, stronger correlations were observed with money lost than with playing frequency. This confirmed the PPAS’s convergent validity. This may reflect ambivalent gambling leads to the unintentional repetitive incurrence of losses.

#### Discriminant validity

No significant correlation was found between PPAS scores and demographic factors, except being younger. This may be a reflection of the instability in the self-identity of young people. For that reason, when researching young people, one needs to be cautious about their overestimation of themselves. Meanwhile, a negative correlation with social desirability was found. A possible explanation for these results is that ambivalent people are susceptible to anxiety because they become introspective in response to reality. To avoid anxiety, a denial mechanism serves to protect them from a negative self-image and, as a result, they tend to answer based on unrealistic images of themselves. In sum, some of the responses to the scale may be biased. On the other hand, similar results have been reported for the SOGS, suggesting that this may be a limitation of self-administered scales (Kuentzel et al. 2008). Therefore, depending on the situation, use of a social desirability scale may be necessary when using the PPAS.

### The utility of the PPAS

While this study showed that some caution may be required when using the PPAS, its reliability and validity were ascertained. Further, the PPAS’s scores showed that the degree of ambivalence correlated with the scores of the DSM5 as the comprehensive severity-assessment scale. Therefore, this study revealed that ambivalence as measured by the PPAS may reflect a core aspect of the condition of a gambling disorder patient. Namely, the PPAS can be considered a useful measure for the assessment for gambling disorders.

### Limitations and suggestions for further research

The recruitment of participants for this study was limited to people registered with an online survey company. As a result, the sample may have been biased and not representative of the general population of pachinko or pachi-slot players in Japan. However, the study’s sample may be considered appropriate, overall, because it consisted mainly of married, middle-class, middle-aged men, which is consistent with the characteristics of most Japanese people who are diagnosed with gambling disorder (Komoto 2014; Toyama et al. 2014). Moreover, participation was limited to people who had played pachinko or pachi-slot only within the previous year. Next, we selected PPAS’s items by not statistic method but specialists’ conferences. As result, inclusion criteria of scale items somewhat became arbitrary. Additionally, to better understand the efficacy of ambivalence to predict prognosis, longitudinal studies are needed. While acknowledging these limitations, the further development and validation of this ambivalence scale for gambling disorder, for use in clinical settings, is recommended.

## Abbreviations

AEQ: Ambivalence over Emotional Expressiveness Questionnaire
AGFI: adjusted goodness of fit index
AGRI: Alberta Gaming Research Institute
AIC: Akaike Information Criterion
CFI: comparative fit index
DSM-5: Diagnostic and Statistical Manual of Mental Disorders-5
GFI: goodness of fit index
G-SAS: Gambling Symptom Assessment Scale
PGSI: Problem Gambling Severity Index
PG-YBOCS: Yale-Brown Obsessive Compulsive Scale-modified for Pathological Gambling
PPAS: Pachinko/Pachi-Slot Playing Ambivalence Scale
RMSEA: root mean square error of approximation
SIRI: Short Interpersonal Reactions Inventory
SOGS: South Oaks Gambling Screen
